# A Two-Stage Automatic System for Detection of Interictal Epileptiform Discharges from Scalp Electroencephalograms

**DOI:** 10.1523/ENEURO.0111-23.2023

**Published:** 2023-11-16

**Authors:** Xiaoyun Wang, Xing Wang, Chong Wang, Zhongyuan Wang, Xiangyu Liu, Xiaoling Lv, Ying Tang

**Affiliations:** 1 Department of Neurology, Nanjing Drum Tower Hospital, The Affiliated Hospital of Nanjing University Medical School, Nanjing 210008, People’s Republic of China; 2 Department of Signal Processing Research, Beijing Solar Electronic Technologies Company Ltd, Beijing 100044, People’s Republic of China; 3 Department of Neurosurgery, Nanjing Drum Tower Hospital, The Affiliated Hospital of Nanjing University Medical School, Nanjing 210008, People’s Republic of China; 4 Geriatrics Research Institute of Zhejiang Province, Zhejiang Provincial Key Lab of Geriatrics, Zhejiang Hospital, Hangzhou 310013, People’s Republic of China; 5 Geriatrics Research Institute of Zhejiang Province, Zhejiang Provincial Key Lab of Geriatrics, Zhejiang Hospital, Hangzhou 310013, People’s Republic of China

**Keywords:** automatic interpretation, deep learning, electroencephalograms, interictal epileptiform discharges, temporal convolutional network

## Abstract

The objective of this work was to develop a deep learning-based automatic system with reliable performance in detecting interictal epileptiform discharges (IEDs) from scalp electroencephalograms (EEGs). For the present study, 484 raw scalp EEG recordings were included, standardized, and split into 406 for training and 78 for testing. Two neurophysiologists individually annotated the recordings for training in channel-wise manner. Annotations were divided into segments, on which nine deep neural networks (DNNs) were trained for the multiclassification of IED, artifact, and background. The fitted IED detectors were then evaluated on 78 EEG recordings with IED events fully annotated by three experts independently (majority agreement). A two montage-based decision mechanism (TMDM) was designed to determine whether an IED event occurred at a single time instant. Area under the precision–recall curve (AUPRC), as well as false-positive rates, F1 scores, and kappa agreement scores for sensitivity = 0.8 were estimated. In multitype classification, five DNNs provided one-versus-rest AUPRC mean value >0.993 using fivefold cross-validation. In IED detection, the system that had integrated the temporal convolutional network (TCN)-based IED detector and the TMDM rule achieved an AUPRC of 0.811. The false positive was 0.194/min (11.64/h), and the F1 score was 0.745. The agreement score between the system and the experts was 0.905. The proposed framework provides a TCN-based IED detector and a novel two montage-based determining mechanism that combined to make an automatic IED detection system. The system would be useful in aiding clinic EEG interpretation.

## Significance Statement

This work has presented a deep learning-based system with a false positive of 0.194 per minute (11.64/h) for sensitivity = 0.8 on 78 whole clinical EEG recordings. These recordings were especially selected to challenge the system, and we therefore would expect better performance in a more general diagnostic scenario. We collected a sizable multi-institute dataset, and the 78 whole clinical EEG recordings for testing have been fully annotated by experts. We believe disclosure of this dataset would benefit research in this field. Additionally, the DNNs were trained for the multiclassification of IED, artifact, and background waveforms. By using this procedure, we attempt to not only improve performance, but also to make a step forward to the ultimate automatic EEG interpretation.

## Introduction

Electroencephalography provides a useful tool for diagnosing neurologic conditions especially epilepsy and other neurologic disorders. Interictal epileptiform discharges (IEDs) are important findings in human EEGs. Their presence strongly supports a diagnosis of epilepsy or an elevated risk of seizures, and their morphologic characteristics and spatial distribution assist in localizing potential foci of seizure origin or in establishing a syndromic diagnosis (Hughes, 1989; Kural et al., 2020a,b). Thus, it is important to determine the existence and location of IEDs in an EEG recording. In current clinical applications, the identification of IEDs requires trained neurologists to interpret EEG recordings through visual inspection and manual annotation. However, it is quite challenging and error prone since the morphologies of IEDs vary and can resemble waves in normal background activity or artifacts (Acharya and Acharya, 2019). It is also time consuming to manually annotate IEDs thoroughly, particularly in EEGs recorded over hours or days. In addition, inter-rater agreement regarding the identification of IEDs is imperfect, leading to the incorrect and delayed diagnoses (Bagheri et al., 2017). In addition, experienced neurologists are in short supply, making EEG services unavailable in much of the world. Therefore, automatic detection of IEDs is highly desirable.

In recent decades, various approaches ranging from mimetic methods to deep learning techniques for automated IED detection in scalp EEG recordings have been proposed (da Silva Lourenço et al., 2021). In general, the machine learning-based detection algorithms often consist of the following three main steps: preprocessing, features extraction, and classification (Abd El-Samie et al., 2018). Because of the natural temporal ordering of EEG signals, EEG classification, the prerequisite for IED detection, can be cast as a time series classification (TSC) problem in which deep neural networks (DNNs) have seen successful applications in the past years (Ismail Fawaz et al., 2020). DNNs can detect latent structures or features from the raw data, thereby reducing the dependence on hand-crafted features. Novel deeper architectures such as convolutional neural networks (CNNs) have achieved high efficiency for epilepsy diagnosis and monitoring (Acharya et al., 2018; Thomas et al., 2018; Tjepkema-Cloostermans et al., 2018; Ansari et al., 2019; Fürbass et al., 2020; Jing et al., 2020; Lin et al., 2020; Thomas et al., 2020; Fürbass et al., 2021).

For instance, in a previous study by Jing et al. (2020), a two-dimensional CNN (2DCNN) for IED detection was proposed and applied for EEG classification. In a parallel work to this study, Thomas et al. (2018) reported a one-dimensional CNN (1DCNN) algorithm for IED detection, achieving a false positive of 2.38/min for a sensitivity of 0.8. They later improved the IED detection system and made remarkable progress to achieve a fivefold cross-validation false positive of 0.2/min (Thomas et al., 2020). However, when performed on a separate testing dataset consisting of 200 30 s scalp EEG segments, the proposed IED detector provided a false positive of 1.43/min for sensitivity of 0.8. Fürbass et al. (2020) described an algorithm based on the structure of fast region-based CNN for automatic detection of IEDs. During a later validation procedure using EEG recordings from 73 patients, they showed that the average per patient false positive of the proposed algorithm was 5.65/h for a sensitivity of 0.92 with respect to clinical template annotations of patients’ typical IEDs (Fürbass et al., 2021).

To implement an automated IED detection system able to aid clinical EEG interpretation, we consider that other most recent DNN architectures achieving state-of-the-art performance in TSC tasks would be trained, tested, and evaluated. In addition, identification of artifacts is one tricky problem in EEG interpretation both manually and automatically. In previous studies, a simple rejection technique is generally applied to filter out high-amplitude artifacts, and detectors are therefore trained for binary classification of IEDs and non-IEDs (Thomas et al., 2018; Jing et al., 2020; Thomas et al., 2020). However, this simple method can lead to insufficient filtration of artifacts and simultaneous rude filtration of spikes that resemble artifacts, adversely affecting the quality of data for training the IED detectors. Novel methods for automatic removal of artifacts from EEG recordings have been proposed, but an important factor for the usability of such artifact removal algorithms is its computational burden (Hartmann et al., 2014). Therefore, we expect that using a more sophisticated dataset containing artifacts for training would improve the performance of IED detectors.

In the current study, we first trained and assessed nine DNN-based IED detectors for EEG classification and IED recognition. Then we applied a two-stage framework to develop the automatic IED detection system. In stage 1, we ran the fitted IED detectors on raw EEG recordings in earlobe and longitudinal bipolar montages to generate a channel-wise probability array of IED at single instant of time. In stage 2, we designed a novel two montage-based decision mechanism (TMDM) for the final determination of an IED event. Our approach presented a temporal convolutional network (TCN)-based system with high performance on detecting IEDs from scalp EEG recordings.

## Materials and Methods

### Participants and scalp EEG recordings

From six clinical institutes, we collected 3840 scalp EEG recordings acquired according to the international 10–20 electrode system during clinical care between 2013 and 2020. Among these recordings, 400 contained IEDs. From these recordings, we excluded 88 that had status epilepticus (SE), electrical SE during sleep (ESES) periodic discharges, and burst suppression, and included all of the remaining 312 recordings. The other 3440 recordings that contained no IEDs during the whole EEG recording session were included randomly at a ratio of 5%. At this point, we included 484 scalp EEG recordings as our final data for the investigation.

Three different EEG acquisition systems were used among institutes. To standardize data, the raw EEG recordings were resampled to 256 Hz and saved in the European Data Format (EDF). After standardization, recordings were split into 406 for training and 78 for testing as described below.

The study was approved by each Institutional Review Board with a waiver of written informed consent from the patients since it involved a retrospective analysis of anonymized data.

### EEG recordings for testing

To challenge the proposed system, by design we selected 78 EEG recordings (57 were with IEDs) for the use of testing and evaluation. The recordings were from 78 patients, and contained (1) IEDs with varying frequencies, morphologies, and spatial distributions; (2) artifacts including movement artifacts, electrocardiogram (ECG) artifacts, and electromyogram (EMG) artifacts; and (3) nonepileptiform transients including, for example, vertex sharps, sleep spindles, and K-complexes.

The duration range of these recordings (ignoring the regions of ictal epileptiform discharges) was between 30 min and 12 h, with a total length of 5657 min and an average length of 72.5 min. Of these recordings, 47 were from clinical routine and 31 from ICU.

The etiology of the epilepsy was as described below. Among the 57 patient EEG recordings with IEDs, 12 showed generalized epilepsy, 22 showed focal epilepsy, 3 showed combined generalized and focal epilepsy, and 20 showed unknown epilepsy. All 21 patient EEG recordings without IEDs did not show epilepsy.

The remaining 406 EEG recordings (255 were with IEDs) were for training. Of these recordings, 184 were from clinical routine and 222 were from ICU.

### Gold standard EEG annotation and dataset assembly

To assemble the training dataset, two expert neurophysiologists independently reviewed and performed annotations on EEG recordings in both earlobe and longitudinal bipolar montages with the aid of the Solar 2848 platform (Solar Electronic Technologies Company Ltd.). Typical IEDs were annotated based on the criteria for definition (Kural et al., 2020a,b) in channel-wise manner. The same two experts annotated single-channel artifacts as well as backgrounds at random locations. Experts were not required to review each recording completely. However, experts were requested to annotate as many distinct waveforms as possible. A degree of mistakes was allowed in the training dataset. Therefore, no agreement between experts was needed, and annotations (i.e., single-channel IEDs, artifacts, or backgrounds) by either expert were included for investigation. Overall, 257,655 annotations were labeled by experts (Extended Data Table 2-1).

Then, annotated recordings were preprocessed by applying a Butterworth high-pass filter of 0.5 Hz, a low-pass filter of 45 Hz, and a low-pass and a notch filter of 50 Hz. After that, each annotation was divided into consecutive 1 s segments with 0.5 s overlap. For those <1 s (basically, IEDs), 1 s segments were extracted with labeled annotations located in the middle. At this point, a dataset consisting of 326,546 labeled segments was assembled (Extended Data Table 2-2).

To assemble the detection dataset, three experts individually performed the annotation of IED events in an exhaustive and time-wise manner on the 57 EEG recordings containing IEDs for testing. During a single IED event, IED waveforms could appear on multiple channels. Events that had a time interval <1 s were treated as one. At last, only IED events with majority agreement (greater than or equal to two of three experts) were labeled. No annotation was performed on the other 21 recordings without IEDs.

### DNN architecture overview

Nine DNNs with different characteristics and architectural elements were trained to perform EEG classification and IED recognition. Structures of each of the nine DNNs are illustrated in Extended Data Figure 1-1.

The multilayer perceptron (MLP) constitutes the simplest and most traditional architecture for deep learning models and was proposed as a baseline architecture for TSC (Ismail Fawaz et al., 2020). However, one impediment to adopting MLP for time series data are that temporal information is lost and features learned are no longer time invariant (Ismail Fawaz et al., 2020).

A fully convolutional network (FCN) is mainly a convolutional network that does not contain any local pooling layers, which means that the length of a time series is kept unchanged throughout the convolutions (Ismail Fawaz et al., 2020). One of the main characteristics of this architecture is the replacement of the traditional final form with one fully connected to a global average pooling (GAP) layer, which reduces drastically the number of parameters in a neural network (Zhou et al., 2015).

Multivariate long short-term memory (MLSTM)-FCN is a multivariate time series classification model developed from LSTM-FCN by augmenting the fully convolutional block with a squeeze-and-excitation block to further improve accuracy (Karim et al., 2019).

CNN is the most widely applied architecture for the TSC problem, which is probably because of their robustness and the relatively small amount of training time compared with complex architectures such as MLP (Ismail Fawaz et al., 2020). Several IED detectors based on 1DCNN and 2DCNN have been developed in previous studies as discussed above. In the present study, we adopted several other variants of CNNs, including ResNet (residual network), InceptionTime, XceptionTime, and TCN for IED detection.

ResNet plays an important role in recent classification tasks. The main characteristic of ResNet is the shortcut residual connection between consecutive convolutional layers, which enhances the accuracy of the model and makes training a DNN much easier by reducing the vanishing gradient effect (Goldberg, 2016). In addition to the basic ResNet, we also used the ResNet50 with one dimension (xresnet1d50) for the task of IED detection.

InceptionTime is an ensemble deep learning model for TSC, which created by cascading multiple Inception modules (Ismail Fawaz et al., 2019). Each individual module will have the same architecture but with different randomly initialized weight values. The core idea of an Inception module is to apply multiple filters simultaneously to an input time series.

XceptionTime is designed to capture both temporal and spatial information by integration of depth-wise separable convolutions, adaptive average pooling, and a novel nonlinear normalization technique (Rahimian et al., 2019). This network is less prone to overfitting and more robust to temporal translation of the input, and more importantly is independent from the input window size.

A TCN is developed by integration of modern convolutional architectures to a one-dimensional FCN with the following two distinguishing characteristics: (1) the convolutions in the architecture are causal, meaning that there is no information “leakage” from future to past; and (2) the architecture can take a sequence of any length and map it to an output sequence of the same length (Bai et al., 2018).

A multilevel wavelet decomposition network (mWDN) is proposed for building frequency-aware deep learning models for time series analysis with the advantage of multilevel discrete wavelet decomposition in frequency learning while enabling the fine-tuning of all parameters under a deep neural network framework (Wang et al., 2018).

### Training of EEG classifiers

The IED, artifact, and background segments constituting the training dataset were input to the DNN models as one-dimensional arrays (256 sample points). Then each network was trained to identify whether a single-channel segment is an IED, an artifact, or a background by calculating the probability values.

The EEG classifiers (IED detectors) were trained and evaluated using the fivefold cross-validation method. The entire dataset of segments was divided into five folds at random by keeping the labels and montage distribution consistent over the different folds. To prevent class imbalance, we applied a resample module to balance the different segment types. The hyperparameters of each network were optimized in a brute force manner. We applied the one-cycle policy application programming interface (Smith and Topin, 2017) to train the models quickly as well as to accomplish more robust convergence.

### Two-stage framework

**Figure 1.**
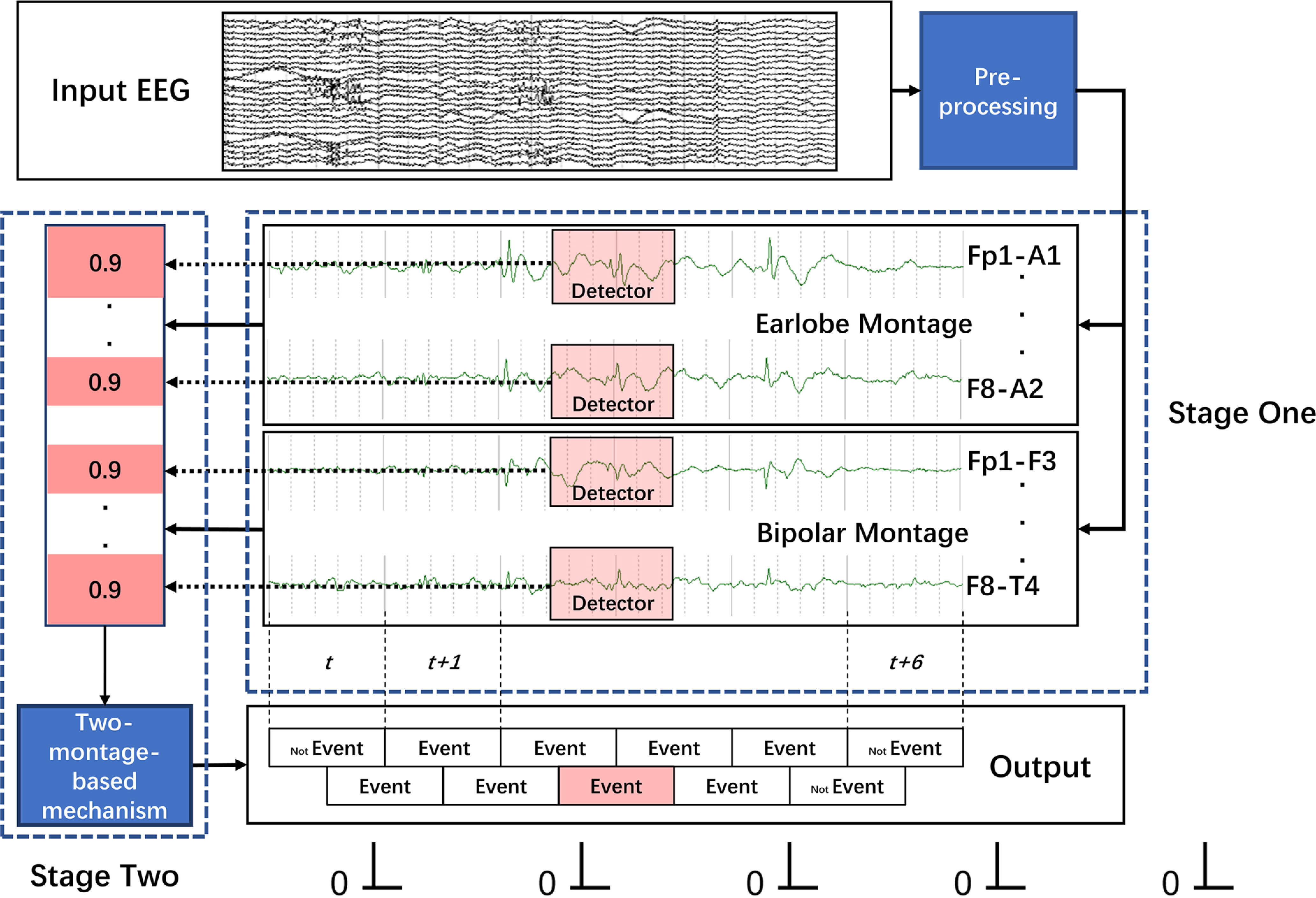
Diagram of the two-stage framework for developing the IED detection system. An example of a 6 s EEG is presented. Structures of each of the 9 DNNs are illustrated in Extended Data Figure 1-1. The two stages refer to the first IED detection stage and the second two montage-based decision stage. In stage 1, the fitted DNN (i.e., detector) is run through EEG recording in channel-wise manner to generate 2 × 19 probability arrays as output. In stage 2, the arrays are put into the two montage-based decision mechanism to produce a final determination whether an IED event occurred. *t* indicates time instant.

IED detection systems were developed following a two-stage framework, as illustrated in Figure 1. The authors operating the system were blinded to all expert annotations.

10.1523/ENEURO.0111-23.2023.f1-1 Figure 1-1 DNNs architectures. Download Figure 1-1, TIF file.

First, EEG recordings were preprocessed by applying a Butterworth high-pass filter of 0.5 Hz, a low-pass filter of 45 Hz, and a low-pass and a notch filter of 50 Hz, and then were input to IED detectors in an earlobe montage and a longitudinal bipolar montage in parallel in a channel-wise manner.

Each of the fitted DNNs with a sliding window of 1 s length (i.e., detectors) was run with a 0.5 s stride. For a single instant of time, detectors generated a two-dimensional array (2 montages × 19 channels), with each value representing the probability of IED for the respective channel of each montage. The probability array was then put into a decision mechanism to produce a determination about whether an IED event occurred at this instant of time.

The decision mechanism borrows the experience of clinical EEG interpretation that integrates multiple montage information (Kane et al., 2017). In usual cases, an occurrence of an IED event may lead to at least one presentation of IED on channels in an earlobe montage and two presentations in a bipolar montage, respectively. Therefore, the first necessary condition to identify an occurrence of an IED event is ≥1 of the 19 probability values from the earlobe montage, and meanwhile, ≥2 of the 19 probability values from the bipolar montage are above an indicated threshold. The second necessary condition is that IEDs present in an earlobe montage should be correlated spatially with that present in a bipolar montage.

As a comparison, the single montage-based decision mechanism (SMDM) was evaluated by considering the maximum of the 19 probability values generated from the earlobe montage. When the maximum value is above a threshold, an IED event is identified.

### Performance assessment and statistical analysis

We calculated the confusion matrices and the overall true positive rates to summarize the results of estimating EEG classifiers by applying the same pipeline throughout the fivefold cross-validation process. We especially evaluated the detectors on IED recognition by assessing the precision–recall (PR) curves via a one-versus-rest approach.

When evaluating the performance of different detection systems, a true-positive detection was defined if the overlap between the detection result and the expert label reaches ≥50%. The PR curves were created for the independent testing dataset to assess the area under the curve (AUC) of the systems to detect IED events. The 95% confidence intervals (CIs) for the area under the PR curve (AUPRC) were computed using bootstrapping with 1000 iterations. The false-positive rates (FPRs) for a fixed sensitivity of 0.8 were calculated for comparison with the different methods proposed in the literature. We also calculated the F1 scores, as well as the Cohen’s Kappa agreement scores between the detection systems and the experts. To calculate the Cohen’s Kappa scores, we generated two binary arrays in relation to the occurrence of IED event per 1 s epoch based on the annotations made by experts or our system. In this context, a value of 0 represents the absence of an IED event in the epoch. Then we used the sklearn.metrics.cohen_κ_score interface to compute the Kappa scores for the two arrays. The probability threshold for the fixed sensitivity of 0.8 was later used in the patient-wise assessment and external evaluation.

Network parameters, inference time under batch size 100,000 with sufficient computational resources (Ryzen 5 3600 6-Core Processor 3.6 GHz, RAM 48.0 GB, and RTX 3070 8 GB GPU, AMD), and detection time for 1 h EEG were measured as the computational efficiency of systems to evaluate their applicability in the clinic, where computational resources could be insufficient.

Patient-wise efficiency of the detection system with outstanding performance was assessed. In brief, the 78 EEG recordings for evaluation were divided into subgroups with varying frequencies of IED events. Measures included the sensitivity, precision, and FPR.

External evaluation was performed on a public dataset (Kural et al., 2020a). The set consists of 100 interictal EEG epochs 10–20 s long, classified by the clinical diagnostic reference standard as having (*n* = 54) or not having (*n* = 46) IEDs. As EEG epochs in earlobe montage were not provided by the dataset, we used the common average and the longitudinal bipolar montage in the following evaluation. Network parameters, probability threshold, and decision mechanism (TMDM) are used in the same way as in the patient-wise assessment. The 95% CIs were obtained using bootstrapping with 5000 replicates.

### Data availability

For all programming, we used Python. For the application of deep learning techniques, we used Pytorch 1.7.1 and the fastai deep learning library 2.2.7. For data preprocessing and manipulation, we used Scikit-Learn 0.24.1, SciPy 1.3.1, and NumPy 1.17.4. The source code was available at https://github.com/opop08/scalp_EEG_classification.

## Results

**Figure 2.**
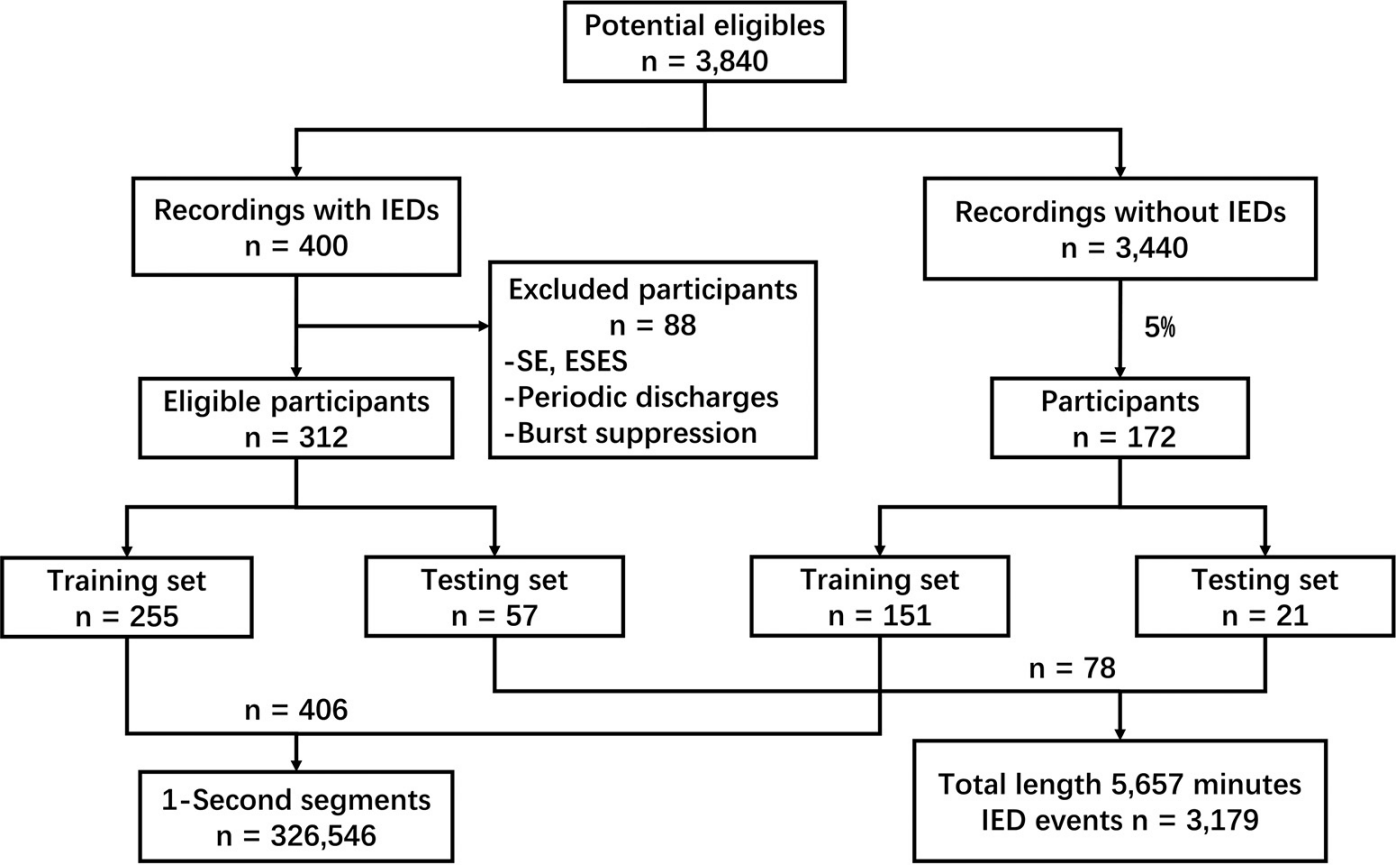
Diagram of data collection and selection. ESES, electrical status epilepticus during sleep. Numbers of annotations by neurophysiologists for training is presented in Extended Data Tables 2-1 and 2-2.

We included and analyzed 484 scalp EEG recordings (312 were with IEDs; Fig. 2) from 361 patients. Characteristics of EEG recordings (age range and sex distribution) are described in Table 1.

**Table 1:** Characteristics of EEG recordings used in training and testing of systems (male/female)

Age range (years)	EEGs with no IEDs	EEGs with IEDs						Total
	A (a)	A (a)	B (b)	C (c)	D (a)	E (a)	F (a)	
406 EEGs from 283 patients used for training								
0–5	2/1	0	0	0	1/4	2/0	1/2	6/7
5–18	11/11	11/17	4/5	0	11/1	5/6	4/2	46/42
18–35	27/26	24/16	19/11	6/2	3/5	0/2	0/3	79/65
35–50	18/20	11/11	4/3	3/6	0/1	0	0/1	36/42
50–70	20/9	4/7	3/3	7/7	1/1	0/1	1/1	36/29
>70	3/3	5/1	0	1/2	1/0	0/1	0/1	10/8
Total	151	107	52	34	29	17	16	406
78 EEGs from 78 patients selected for testing								
0–5	0/1	0	1/0	0	0	0	0	1/1
5–18	4/2	3/3	1/1	1/0	2/2	2/1	0/1	13/10
18–35	7/3	3/4	1/5	0/1	0	1/3	3/1	15/17
35–50	1/0	1/3	1/2	1/1	0	0	1/0	5/6
50–70	1/1	2/0	1/1	0	0	0	0	4/2
>70	0/1	0	0/2	1/0	0	0	0	2/3
Total	21	19	16	5	4	7	6	78

A–F indicate the 6 institutes providing raw EEG recordings: A, The Affiliated Drum Tower Hospital of Nanjing University; B, The Second Affiliated Hospital of Zhejiang University; C, Guizhou Provincial People’s Hospital; D, The First Affiliated Hospital of Nanchang University; E, The Affiliated Hospital of Xuzhou Medical University; F, Yan’an People’s Hospital. a–c indicate three different EEG acquisition equipment provided by manufacturers: a, Solar Electronic Technologies Company Ltd.; b, NIHON KOHDEN; c, Natus.

**Table 2:** Evaluation of systems based on different detectors and decision mechanisms

Detectors	AUC (95% CIs)		False positive/min	F1 score	Kappa score
	TMDM	SMDM	TMDM	TMDM	TMDM
MLP	0.622 (0.497– 0.724)	0.568 (0.442–0.671)	0.740	0.512	0.806
FCN	0.668 (0.580–0.748)	0.599 (0.503–0.680)	1.178	0.493	0.642
TCN	0.811 (0.723–0.873)	0.738 (0.648–0.812)	0.194	0.745	0.905
ResNet	0.759 (0.677–0.825)	0.690 (0.591–0.778)	0.324	0.672	0.880
xresnet1d50	0.786 (0.704–0.848)	0.710 (0.623–0.780)	0.199	0.742	0.905
InceptionTime	0.802 (0.715-0.867)	0.746 (0.658–0.818)	0.223	0.728	0.904
XceptionTime	0.812 (0.738–0.887)	0.737 (0.637–0.849)	0.205	0.739	0.908
MLSTM	0.776 (0.689–0.843)	0.724 (0.632–0.799)	0.374	0.657	0.870
mWDN	0.692 (0.586–0.784)	0.620 (0.510–0.713)	0.226	0.726	0.841

TMDM and SMDM indicate different decision mechanisms.

10.1523/ENEURO.0111-23.2023.tab2-1 Table 2-1 Numbers of annotations by neurophysiologists for training. Two expert neurophysiologists independently reviewed and performed channel-wise annotations on EEG recordings in both earlobe and longitudinal bipolar montages with the aid of the Solar 2848 platform. Agreement between experts is not required. Download Table 2-1, DOCX file.

10.1523/ENEURO.0111-23.2023.tab2-2 Table 2-2 Statistics of 1 s segments for network training. The annotated recordings are preprocessed and divided into consecutive 1 s segments with 0.5 s overlap. Download Table 2-2, DOCX file.

### EEG classifiers based on DNNs

**Figure 3.**
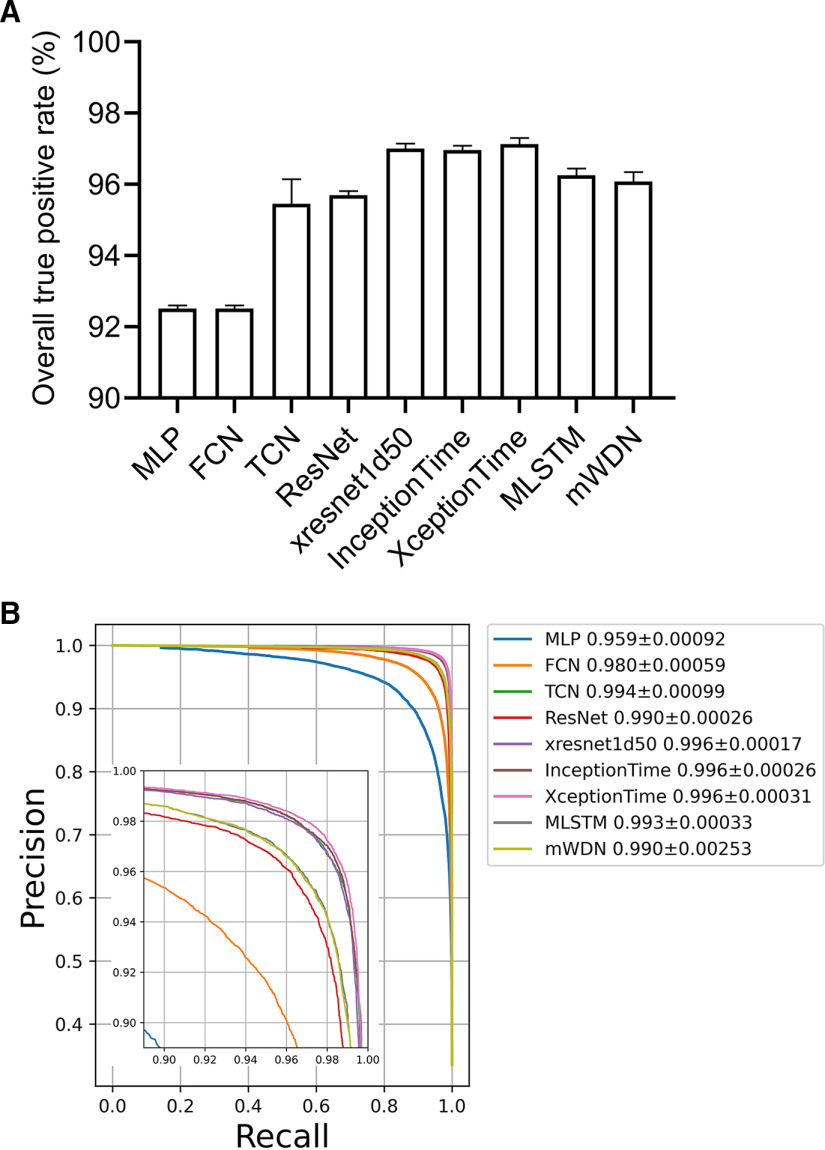
Evaluation of EEG classifiers. ***A***, Overall true-positive rate of different DNN-based classifiers on multiclassification of EEG segments. Values are calculated from mean numbers over the fivefold cross-validation. ***B***, The fivefold cross-validation precision–recall curves using one-versus-rest approach. The mean ± SD AUC values are presented. Confusion matrices are presented in Extended Data Figure 3-1.

From the fivefold cross-validation confusion matrices (Extended Data Fig. 3-1), overall true-positive rates are calculated and presented in Figure 3*A*. The EEG classifiers based on xresnet1d50, XceptionTime, and InceptionTime provide the best performance, achieving mean ± SD overall true-positive rates of 97.00 ± 0.14, 97.13 ± 0.18, and 96.96 ± 0.13, respectively. TCN (95.45 ± 0.70), ResNet (95.69 ± 0.13), MLSTM (96.26 ± 0.19), and mWDN (96.08 ± 0.27) also exhibit well.

10.1523/ENEURO.0111-23.2023.f3-1 Figure 3-1 The fivefold cross-validation confusion matrices evaluating different classifiers. BKG, Backgrounds; ATF, artifacts. Numbers indicate mean values over 5 pipelines. Download Figure 3-1, TIF file.

10.1523/ENEURO.0111-23.2023.f4-1 Figure 4-1 EEG example of a false detection caused by artifact. The 1.5-second EEG that contains an artifact (marked in black) is falsely annotated by the proposed TCN-based system as an IED event. The waveforms of this artifact resemble IEDs (especially the waveform appears on channel P4-O2 in bipolar montage) and fit the spatial distribution of discharges. Upper is in earlobe montage, and below is in bipolar montage. Download Figure 4-1, TIF file.

10.1523/ENEURO.0111-23.2023.f4-2 Figure 4-2 EEG example of an IED event detected by the TCN-based system but missed by expert neurophysiologists. The 1-second EEG (marked in black) in bipolar montage contains an IED event with low amplitude. IED waveforms on channels P3-O1 and F7-T3 (marked in black rectangles) appear clearly when displayed using an amplitude sensitivity of 5 μV/mm. This IED event is missed by expert neurophysiologists since there is a more obvious IED event nearby. Views using different amplitude sensitivities were presented (upper, 10 μV/mm; below, 5μV/mm). Download Figure 4-2, TIF file.

We applied a one-versus-rest approach to compare the ability of the EEG classifiers to identify an IED waveform. The fivefold cross-validation PR curves are presented in Figure 3*B*, with mean ± SD AUC values calculated and listed in the legend. XceptionTime (0.996 ± 0.00031), InceptionTime (0.996 ± 0.00026), xresnet1d50 (0.996 ± 0.00017), TCN (0.994 ± 0.00099), and MLSTM (0.993 ± 0.00033) are superior to the other four DNNs.

### IED detection systems

IED detection systems based on different detectors and decision mechanisms were evaluated on a separate dataset consisting of 78 whole clinical EEG recordings with a total length of 5657 min (minimum, 30 min; maximum, 12 h; average, 72.5 min) as described in the Materials and Methods section.

The PR curves were created to assess AUC of the systems to detect IED events. As presented in Table 2, systems implementing the two montage-based decision mechanism exhibit superior performance with better AUC values than systems implementing the single montage-based decision mechanism. The TCN-based and XceptionTime-based systems provides the best AUPRCs (TCN: 0.811; 95% CI, 0.723–0.873; XceptionTime: 0.812; 95% CI, 0.715–0.867).

We compared the FPR for a fixed sensitivity value of 0.8 for further evaluating the proposed IED detection systems (Table 2). The systems based on TCN provide the best with a false positive of 0.194/min (i.e., 11.64/h) and F1 scores of 0.745, surpassing other systems.

To evaluate the reliability of systems to detect the IED events at the correct location, the Cohen’s Kappa agreement scores between systems and the experts were estimated. As presented in Table 2, scores for the systems based on TCN, xresnet1d50, XceptionTime, and InceptionTime are >0.9, surpassing other systems.

In addition, the system based on TCN shows higher computational efficiency, as indicated by network parameters, inference time (in milliseconds), and detection time in Table 3. Therefore, TCN would be more suitable for real-time processing and would have better clinical availability.

**Table 3:** Computational efficiency of systems implementing the two-montage-based decision mechanism

Detectors	Parameters (×1,000)	Inference time, ms (batch size = 100,000)	Detection time (s/h EEG)
MLP	59	364	5.39
FCN	140	1447	8.29
TCN	67	3178	11.78
ResNet	479	3018	11.30
xresnet1d50	2122	8410	23.58
InceptionTime	871	5903	18.70
XceptionTime	895	8902	25.89
MLSTM	418	1765	9.07
mWDN	562	3539	13.19

### Patient-wise assessment of TCN-based system

We evaluated the performance of the proposed TCN-based system on individual EEG recordings with varying frequencies of IED events. As shown in Table 4, for 21 EEG recordings containing no IEDs but with distinct types of artifacts and physiological transient variants, false-positive detection is at a patient average of 0.062/min (3.72/h). For 57 EEG recordings with IEDs, false-positive detection is at a patient average of 0.273/min (16.4/h) for an average sensitivity of 0.729. Comparing by groups, sensitivity, and precision tend to be lower, whereas FPR tends to be better in recordings with less frequent IED events. Of five EEG recordings with one IED event per hour, miss detection (i.e., no IED event is detected) is observed in three recordings.

**Table 4:** Patient-wise evaluation of the IED detection system based on TCN

Frequency (IED event/h)	Numbers	Sensitivity	Precision	False positive/min (false positives/h)
0	21			0.062 ± 0.069 (3.72)
1–10	18	61.5 ± 38.8	40.1 ± 39.4	0.092 ± 0.123 (5.52)
10–50	22	70.7 ± 19.7	72.7 ± 18.6	0.151 ± 0.172 (9.06)
50–100	7	67.0 ± 20.7	65.6 ± 13.1	0.387 ± 0.192 (23.2)
>100	10	88.0 ± 7.4	83.4 ± 23.9	0.717 ± 0.804 (43.0)
Subtotal	57	72.9 ± 23.8	66.4 ± 28.8	0.273 ± 0.436 (16.4)

Values indicate mean ± SD or mean.

### False detection analysis

The case-by-case analysis shows that the false detections by the TCN-based system were mainly related to three clusters including artifacts, nonepileptiform physiological events, and real IED events that were missed by expert annotation. Representative examples were shown in Extended Data Figures 4-1 and 4-2.

Artifacts are established causes of error in manual and automatic interpretation of EEG recordings. The TCN-based system was robust to typical artifacts such as movement artifacts and ECG artifacts, but failed to ignore those that resemble IEDs and fit the spatial distribution of discharges (Extended Data Fig. 4-1). Additionally, the system was interfered by normal EEG variants such as benign epileptiform transients of sleep, positive occipital sharp transient of sleep, and some epileptiform vertices.

The IED events that were detected by the TCN-based system but were missed by expert neurophysiologists were mainly events with low amplitude, since the expert neurophysiologists tend to notice more and annotate the IED events with higher amplitude and a typical morphology during the visual inspection of the same recording (Extended Data Fig. 4-2).

### External evaluation

At last, we evaluated the proposed TCN-based system on a public dataset (Table 5). Despite trained for earlobe and bipolar montages, the detector is robust against the common average montage. Precision, sensitivity, and F1 score are 0.679 (95% CI, 0.574–0.785), 0.944 (95% CI, 0.875–0.999), and 0.790 (95% CI, 0.709–0.863), respectively. A system based on the two-montage decision mechanism has a better precision of 0.914 (95% CI, 0.829–0.980) and a good F1 score of 0.840 (95% CI, 0.753–0.909), but a lower sensitivity of 0.778 (95% CI, 0.660–0.885).

**Table 5:** External evaluation of the TCN-based system

Decision mechanism	Montage	Sensitivity (95% CIs)	Precision (95% CIs)	F1 score (95% CIs)
SMDM	Common average	0.944 (0.875–0.999)	0.679 (0.574–0.785)	0.789 (0.709–0.863)
	Bipolar	0.944 (0.875–0.999)	0.698 (0.594–0.797)	0.801 (0.723–0.872)
TMDM		0.778 (0.660–0.885)	0.914 (0.829–0.980)	0.840 (0.753–0.909)

SMDM, Single montage-based decision mechanism (TMDM and SMDM indicate different decision mechanisms). In the common average montage, it requires at least one output value above the threshold for activation. Whereas in the longitudinal bipolar montage, it requires at least two output values above the threshold for activation.

## Discussion

In this study, we presented a novel deep learning-based IED detection system by a developing procedure with the following two primary innovations: a TCN-based detector trained on elaborately annotated dataset for multitype EEG classification; and a new two montage-based decision mechanism for IED event determination. To develop this system, we have used a sizable dataset from six clinical institutes for the whole evaluation. Our final system can provide the channel-wise location of IEDs, which can be applied to EEG recordings with an arbitrary number of channels.

Compared with two similar works (Tjepkema-Cloostermans et al., 2018; Thomas et al., 2020), the currently proposed IED detection system has exhibited superiority. Tjepkema-Cloostermans et al. (2018) have evaluated a 2DCNN-based IED detector that achieved a patient-wise average sensitivity of 0.47 with a false-positive rate of 0.6/min 7 EEG recordings with focal epileptiform discharges. Thomas et al. (2020) have recently proposed a 1DCNN for localizing IEDs in EEG recordings that has achieved a mean fivefold cross-validation AUPRC of 0.838 on their training dataset and a false-positive rate of 1.43/min for a sensitivity of 0.8 on a separate testing dataset consists of 200 30 s scalp EEG segments. Although the dataset used is different, our system provides better performance than these two algorithms: the TCN-based IED detector achieves a mean fivefold cross-validation AUPRC of 0.993 on the training dataset consists of 74 900 IED segments and 251 646 non-IED segments (Fig. 3*B*). In further clinical evaluation test using 78 raw clinical EEG recordings with a total length of 5657 min, the TCN-based IED detection system achieves a false-positive rate of 0.194/min (11.64/h) for a sensitivity of 0.8 (Table 2). In patient-wise assessment, the system shows an average false-positive rate of 0.273/min (16.4/h) with an average sensitivity of 0.729 on 57 EEG recordings containing IEDs.

Our performance is comparable with the IED detection system proposed by Clarke et al. (2021), which is based on 2DCNN and has achieved a mean fivefold cross-validation false-positive rate of 1.16/min for a sensitivity of 0.97. However, this system was not evaluated on an independent dataset. Still, this study mainly focuses on IED detection for patients with idiopathic generalized epilepsy and cannot be directly extrapolated to IED detection performance evaluated against all types of epilepsy. DeepSpike, an algorithm based on the structure of a Fast Region-based CNN for automatic detection of IEDs (Fürbass et al., 2020), has achieved an average per patient false positive rates of 5.65/h for a sensitivity of 0.92 with respect to clinical template annotations of a patient’s typical IEDs (Hartmann et al., 2014; Fürbass et al., 2021), surpassing the present TCN-based system. Nevertheless, the detection procedure (including the patient-wise assessment) of our study has used selected datasets to challenge the performance of the system by including EEG recordings with no IEDs (*n* = 21), as well as recordings containing nontypical IEDs, artifacts, and nonepileptiform transients. We would expect better performance of the system in a more general diagnostic scenario.

We believe that three main reasons, including an EEG annotation strategy, a deep neural network, and a two montage-based decision mechanism, have contributed significantly to the good performance of the proposed system.

The detectors based on TCN, XceptionTime, and InceptionTime present considerably superior performance and preeminent generalization ability compared with the rest based on six other DNNs, achieving (1) one-versus-rest mean AUPRC ≥0.993 on a training dataset (Fig. 3*B*); and (2) AUPRC >0.8 on a testing dataset (Table 2). These DNNs are with novel architectural elements such as dilated convolutions (Yu and Koltun, 2015), residual connections (He et al., 2016), causal convolutions (Oord et al., 2016), and GAP (Lin et al., 2013), which through priori constraints and/or ensemble-like behavior (Laha and Raykar, 2016), may significantly improve the ability of networks to extract latent features of, in the current scenario, EEG waveforms. For instance, the causal convolution is a structure in which an output at time *t* is convolved only with elements from time *t* and earlier in the previous layer. It is remarkably effective in capturing features in sequential signals. Having such an architectural element, TCN has presented an excellent solution in TSC tasks such as electrocardiograph classification, motion sensor-based action recognition, and sleep stage classification (Jia et al., 2020; Mahmud et al., 2020; Stergiou and Poppe, 2020). To the best of our knowledge, this is the first time that TCN has been applied in IED detection based on multiclassification of EEG waveforms. Of importance, the TCN-based system is more computationally efficient compared with InceptionTime and XceptionTime, as indicated by fewer parameters and lower inference time, and takes 11.78 s for loading, preprocessing, and evaluating 1 h of 19-channel raw EEG data (Table 3). Therefore, the TCN-based IED detection system is suitable for the clinic where computational resources would be limited.

Another novelty in the present study is designing a two montage-based decision mechanism for IED determination. This simple mechanism refers to the clinical EEG interpretation that uses several different montages since each montage provides a different perspective on the activity, and inspection of the activity with more than one perspective increases the accuracy of the interpretation (Acharya and Acharya, 2019). In the current study, earlobe and longitudinal bipolar montages were used in combination because of their complementary advantages for the purpose of IED detection. Therefore, the mechanism is interpretable. In brief, the shape and amplitude of waveforms are well preserved, and abnormal waveforms are easy to be recorded and recognized in earlobe montage. However, various sources of artifacts appearing in this montage can interfere with signals, leading to misreading during EEG interpretation. Bipolar montage, on the contrary, has a major advantage of eliminating artifacts. In addition, this montage is useful for analyzing highly localized discharges, particularly when there is a steep potential gradient between adjacent channels. Main weaknesses of bipolar montage include distortion of the shape and amplitude of waveforms, and cancelation of the discharges with a broad field. The superiority of uniting these two montages was evidenced in Table 2 (generally improved AUPRCs) and Table 5 (better F1 score). This decision mechanism is also extendible since more rules relevant to clinical interpretation of EEGs could be put into the decision mechanism. In addition, the two montage-based mode also provide the possibility of further improving performance of the system by applying a “pretraining/fine-tune” strategy to develop respective detectors for montages.

The patient-wise evaluation (Table 4) has revealed a major shortage of the current system: the sensitivity and precision are low with large variabilities in EEG recordings with infrequent IED events (i.e., 1–10 events/h in the present study). This is because the number of true positives in such recordings is small. Therefore, a few false negatives and false positives can lead to an abrupt decrease in sensitivity and precision. In addition, miss detection is observed in three EEG recordings with one IED event per hour. The patient-level results have significance in providing clinical practice instructions. In the clinical interpretation of EEGs, especially those having infrequent IEDs, neurologists need to annotate IED events exhaustively to avoid missed detection and diagnosis. This is the most time consuming and error prone. Therefore, automatic IED detection systems should accomplish high and robust performance in different EEG recordings to fulfill clinical demand. In the case of our system, the detection threshold can be tuned in clinical practice to make a higher sensitivity at the expense of FPR to avoid missed detection.

## Synthesis

Reviewing Editor: William Stacey, University of Michigan Decisions are customarily a result of the Reviewing Editor and the peer reviewers coming together and discussing their recommendations until a consensus is reached. When revisions are invited, a fact-based synthesis statement explaining their decision and outlining what is needed to prepare a revision will be listed below. The following reviewer(s) agreed to reveal their identity: NONE.

Due the delay of the second reviewer in submitting a report and to prevent further delays, we base our decision for revision based on the one reviewer’s comments. The reviewer comment’s are appended. Please revise the manuscript by taking into account all points raised by the reviewer.

Reviewer 1:

Interictal Epileptiform Discharges (IEDs) are hallmarks of EEG recording of patients with epilepsy. Quantifying their occurrence and their spatial distribution is of utmost importance for epilepsy diagnosis. Manually annotating these events is tedious and time consuming. The authors therefore proposed an automated two-stage detection procedure based on deep learning models. The authors have used 406 EEG recordings for training and 78 independent recordings for testing. The results of the 5-fold cross-validation are impressive (area under the precision-recall curve >0.993). For the test dataset, a second stage was added to mimic the human reviewer and utilized two montages. This second stage permits to have a low false detection rate per hour (11.64/h), a good F1 score of 0.752, and a good system-experts agreement of 0.696. The results once reported at a patient-level are, surprisingly, much lower (sensitivity of 69.3{plus minus}26.6 and precision of 61.9{plus minus}29.2) with a large variability. In sum, 40% of the detected events are wrong and 30% are missed in average. This discrepancy, which is acknowledged by the authors but not further discussed, is surprising and may hinder its applicability in clinical routine. Moreover, there is no comparison with previously published detectors run on the same dataset. The aim of the manuscript is sound but some elements of the methods section are unclear and the reported results seem unpractical and current misleading to a naive reader.

Major comments:

1- The quality of the current manuscript would be greatly improved if patient-level results were further discussed.

2- It seems that the authors set the parameters of the detector at 80% of sensitivity based on the results of the test set. All the parameters should be fixed after the training stage, and used as such on the test set. If it is not the case, the current results of the test set are irrelevant due to overfitting.

3- How was the confident interval calculated for the test set? Similar question about the Kappa.

4- The training dataset contained 326,546 annotated segments, randomly split into 5 folds for the cross validation. It seems that segments from the same patients could be in different splits. Could this explain the better results in the training set compared to the test set?

5- The author used an earlobe montage. This EEG montage is, to my knowledge, relatively uncommon. How robust would the detector be against another type of montage?

6- There is no comparison with previously published detectors applied to the same data set. This would provide a baseline for comparison.

Minor comments:

1- The authors mentioned a bipolar montage. There exist several types (e.g., longitudinal, transversal). Please defined in the Methods section which one was used.

2- What does the authors mean by ‘intact’ EEG recording? Raw and continuous?

3- Deep learning was used in the first stage but the second stage is rather empirical. Why did the authors not build a deep learning model which used both montages simultaneously instead of this second stage? Similar question, why is a single channel detection procedure used instead of a multi-channel one?

4- Recording with status epilepticus were excluded. There is surprisingly no mention of seizures in the manuscript. Were they included? If so, were ictal epileptiform discharges used as well? They are generally different from their interictal counterparts.

5- “All the 21 patient EEG recordings without IEDs were non-epilepsy.” Correct this sentence

6- “XceptionTime (0.996), InceptionTime (0.996), xresnet1d50 (0.996), TCN (0.994) and MLSTM (0.993) exhibit superior to the rest four DNNs.” Missing SD values

7- “should be correlated in spatial”, spatially/in space
