# Supplementary figures and images for: A Two-Stage Automatic System for Detection of Interictal Epileptiform Discharges from Scalp Electroencephalograms
Source: eNeuro. 2023 Nov 16;10(11):ENEURO.0111-23.2023. doi: 10.1523/ENEURO.0111-23.2023 (PMC10668214; doi:10.1523/ENEURO.0111-23.2023)

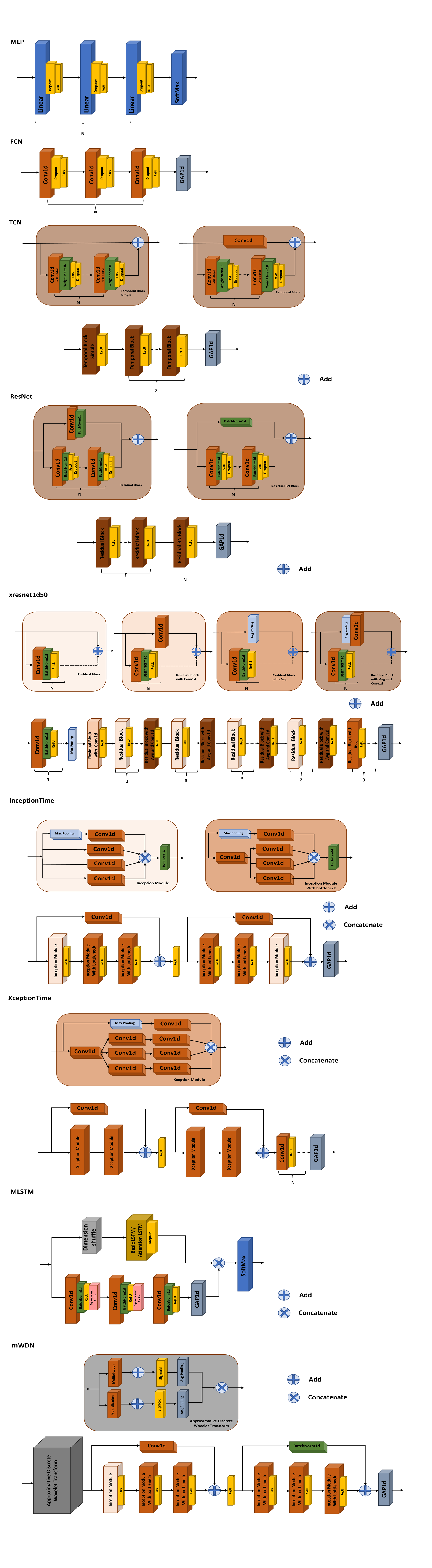

Supplement: Figure 1-1 — DNNs architectures. Download Figure 1-1, TIF file. [file enu-eN-MNT-0111-23-s02.tif]

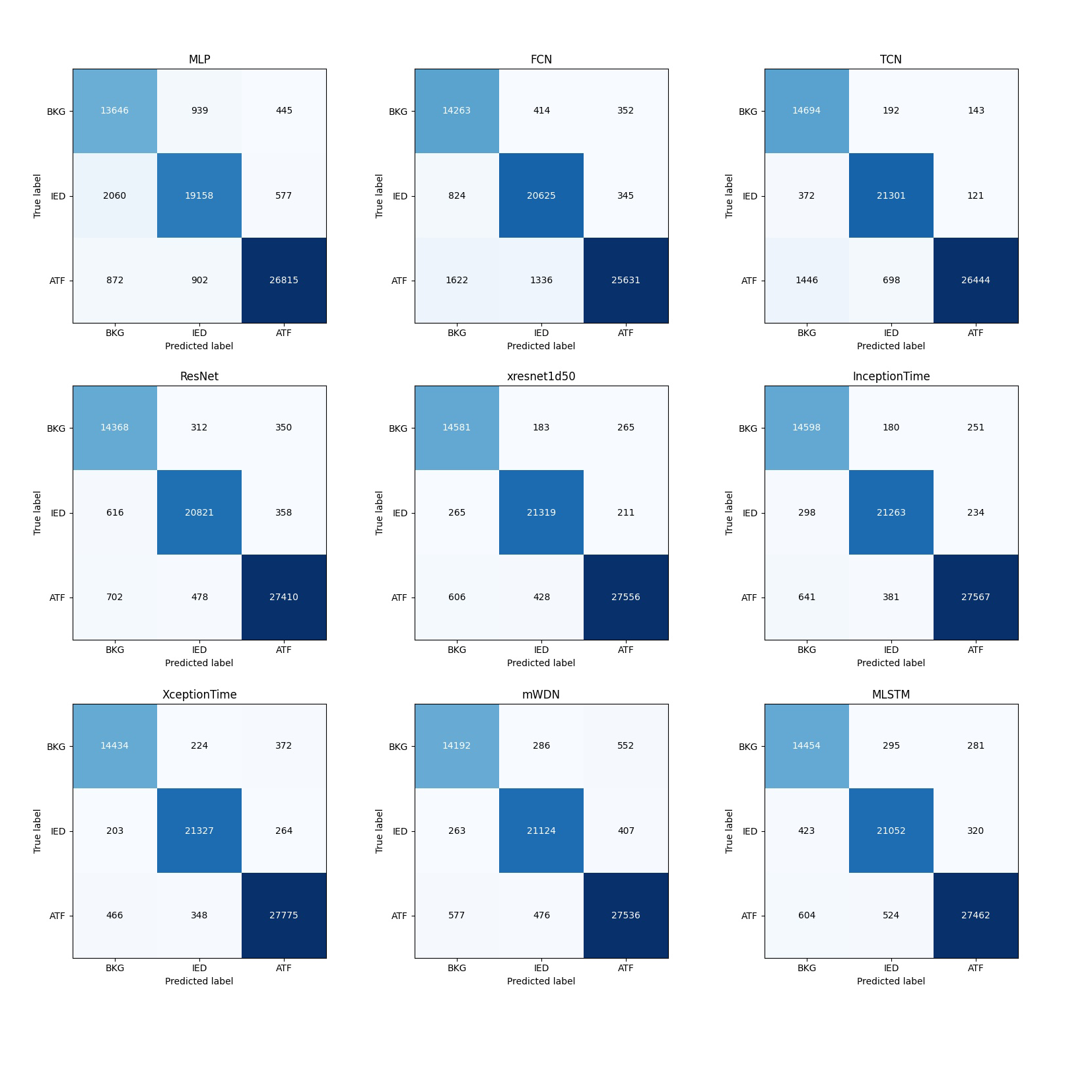

Supplement: Figure 3-1 — The fivefold cross-validation confusion matrices evaluating different classifiers. BKG, Backgrounds; ATF, artifacts. Numbers indicate mean values over 5 pipelines. Download Figure 3-1, TIF file. [file enu-eN-MNT-0111-23-s03.tif]

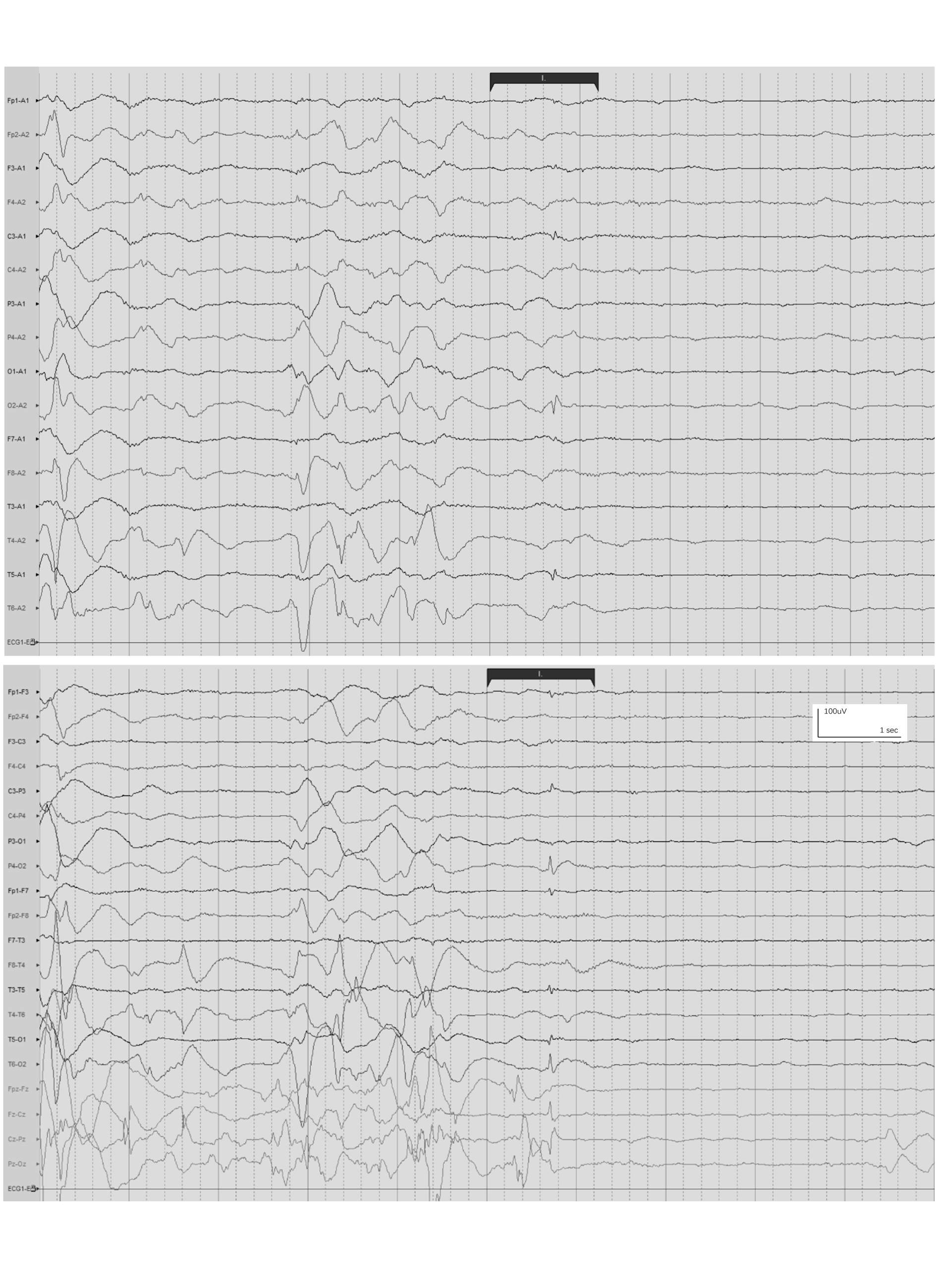

Supplement: Figure 4-1 — EEG example of a false detection caused by artifact. The 1.5-second EEG that contains an artifact (marked in black) is falsely annotated by the proposed TCN-based system as an IED event. The waveforms of this artifact resemble IEDs (especially the waveform appears on channel P4-O2 in bipolar montage) and fit the spatial distribution of discharges. Upper is in earlobe montage, and below is in bipolar montage. Download Figure 4-1, TIF file. [file enu-eN-MNT-0111-23-s04.tif]

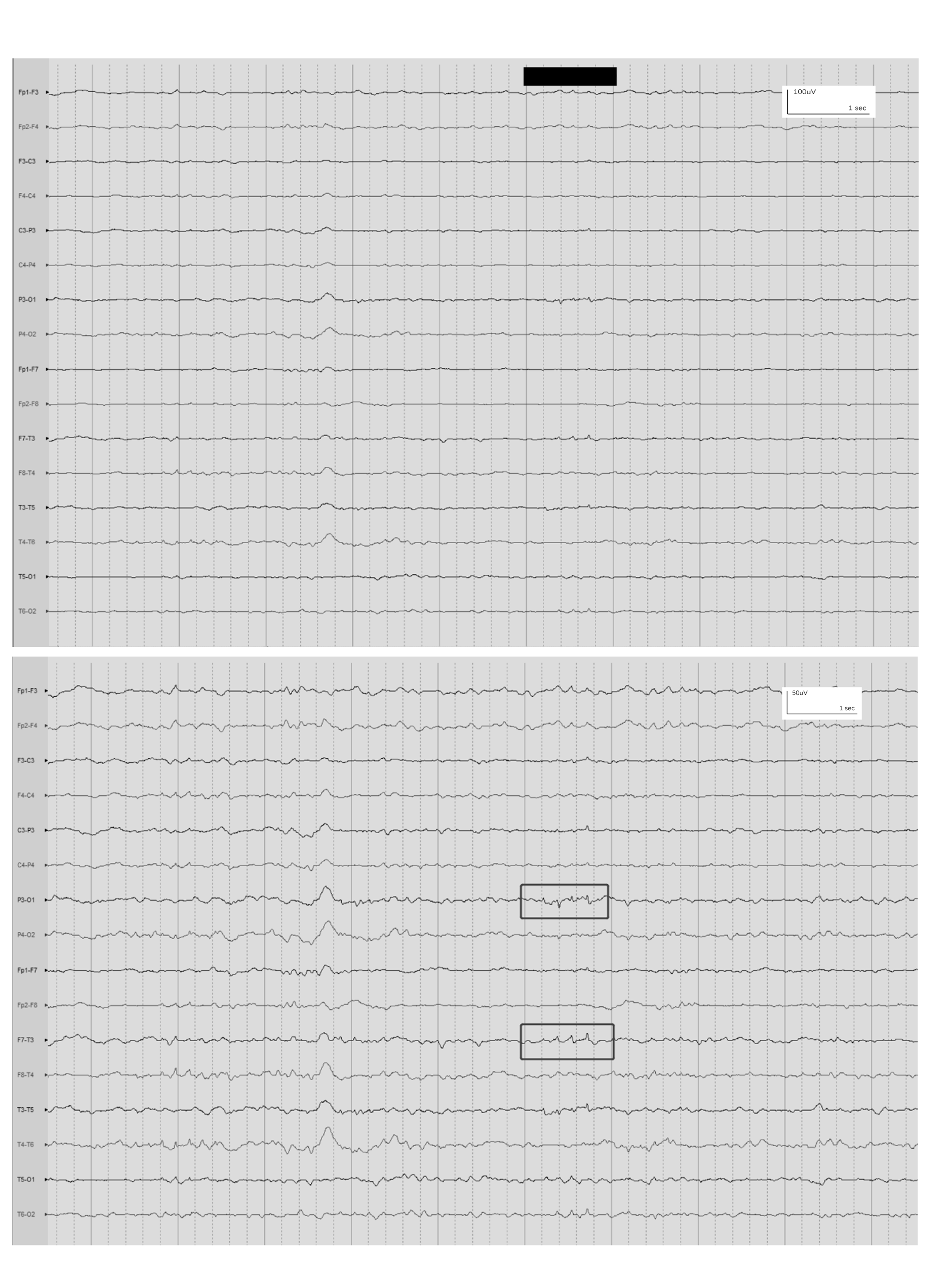

Supplement: Figure 4-2 — EEG example of an IED event detected by the TCN-based system but missed by expert neurophysiologists. The 1-second EEG (marked in black) in bipolar montage contains an IED event with low amplitude. IED waveforms on channels P3-O1 and F7-T3 (marked in black rectangles) appear clearly when displayed using an amplitude sensitivity of 5 μV/mm. This IED event is missed by expert neurophysiologists since there is a more obvious IED event nearby. Views using different amplitude sensitivities were presented (upper, 10 μV/mm; below, 5μV/mm). Download Figure 4-2, TIF file. [file enu-eN-MNT-0111-23-s05.tif]
