# Supplementary material for: A Two-Stage Automatic System for Detection of Interictal Epileptiform Discharges from Scalp Electroencephalograms
Source: eNeuro. 2023 Nov 16;10(11):ENEURO.0111-23.2023. doi: 10.1523/ENEURO.0111-23.2023 (PMC10668214; doi:10.1523/ENEURO.0111-23.2023)
Supplement: Table 2-1 — Numbers of annotations by neurophysiologists for training. Two expert neurophysiologists independently reviewed and performed channel-wise annotations on EEG recordings in both earlobe and longitudinal bipolar montages with the aid of the Solar 2848 platform. Agreement between experts is not required. Download Table 2-1, DOCX file. [file enu-eN-MNT-0111-23-s06.docx]

Extended Table 2-1. Numbers of annotations by neurophysiologists for training.

| Montages | Annotation types | | | Total |
| --- | --- | --- | --- | --- |
|  | Background | IED | Artifact |  |
| Earlobe | 20,716 | 58,405 | 63,971 | 143,092 |
| Bipolar | 14,090 | 47,925 | 52,548 | 114,563 |
| Total | 34,806 | 106,330 | 116,519 | 257,655 |
