# Supplementary material for: A Two-Stage Automatic System for Detection of Interictal Epileptiform Discharges from Scalp Electroencephalograms
Source: eNeuro. 2023 Nov 16;10(11):ENEURO.0111-23.2023. doi: 10.1523/ENEURO.0111-23.2023 (PMC10668214; doi:10.1523/ENEURO.0111-23.2023)
Supplement: Table 2-2 — Statistics of 1 s segments for network training. The annotated recordings are preprocessed and divided into consecutive 1 s segments with 0.5 s overlap. Download Table 2-2, DOCX file. [file enu-eN-MNT-0111-23-s07.docx]

Extended Table 2-2. Statistics of 1-second segments for network training.

| Montages | Segment types | | | Total |
| --- | --- | --- | --- | --- |
|  | Background | IED | Artifact |  |
| Earlobe | 42,205 | 59,642 | 77,697 | 179,544 |
| Bipolar | 32,695 | 49,218 | 65,089 | 147,002 |
| Total | 74,900 | 108,860 | 142,786 | 326,546 |
